# Comparison of High vs. Normal/Low Protein Diets on Renal Function in Subjects without Chronic Kidney Disease: A Systematic Review and Meta-Analysis

**DOI:** 10.1371/journal.pone.0097656

**Published:** 2014-05-22

**Authors:** Lukas Schwingshackl, Georg Hoffmann

**Affiliations:** University of Vienna, Faculty of Life Sciences, Department of Nutritional Sciences, Vienna, Austria; Emory University, United States of America

## Abstract

**Background:**

It was the aim of the present systematic review and meta-analysis to investigate the effects of high protein (HP) versus normal/low protein (LP/NP) diets on parameters of renal function in subjects without chronic kidney disease.

**Methods:**

Queries of literature were performed using the electronic databases MEDLINE, EMBASE, and the Cochrane Trial Register until 27^th^ February 2014. Study specific weighted mean differences (MD) were pooled using a random effect model by the Cochrane software package Review Manager 5.1.

**Findings:**

30 studies including 2160 subjects met the objectives and were included in the meta-analyses. HP regimens resulted in a significantly more pronounced increase in glomerular filtration rate [MD: 7.18 ml/min/1.73 m^2^, 95% CI 4.45 to 9.91, p<0.001], serum urea [MD: 1.75 mmol/l, 95% CI 1.13 to 237, p<0.001], and urinary calcium excretion [MD: 25.43 mg/24h, 95% CI 13.62 to 37.24, p<0.001] when compared to the respective LP/NP protocol.

**Conclusion:**

HP diets were associated with increased GFR, serum urea, urinary calcium excretion, and serum concentrations of uric acid. In the light of the high risk of kidney disease among obese, weight reduction programs recommending HP diets especially from animal sources should be handled with caution.

## Introduction

In face of the worldwide increase in prevalence of obesity, a large number of dietary measures aiming at weight reduction of weight management have been described. These diets differ mainly with respect to macronutrient composition, and among them, a high protein (HP) regimen has gained interest in recent years. However, there is inconsistent data regarding the potential beneficial or detrimental effects of HP diets on parameters of obesity as well as its associated risks. While HP protocols were reported to be advantageous when compared to their low/normal protein counterparts in short-term trials [1], no such benefits on outcome markers of obesity, cardiovascular disease or glycemic control could be reported in a recent meta-analysis investigating long-term interventions [2]. In 2002, the Institute of Medicine published an acceptable macronutrient distribution range (AMDR) for protein of 5–35% of daily calories (depending on age), with a special emphasis that there is insufficient data on the long-term safety of the upper limit of this range [3]. A major concern in relation to potential deleterious effects of HP diets is the increased risk of renal dysfunction [4], [5]. High protein intake is regarded to be a trigger of renal hyperfiltration and may therefore cause renal damage [6]. In animal and human studies, HP consumption has been found to accelerate chronic kidney disease (CKD), raise albuminuria and diuresis, natriuresis, and kaliuresis [7]. Epidemiological data from the Nurses' Health study showed that high intake of non-dairy animal protein may accelerate renal dysfunction in women with an already established mild renal insufficiency (glomerular filtration rate (GFR) <80 ml/min/1.73 m^2^), while HP intake was not associated with a decline in regular renal function in women (initial GFR values > 80 ml/min/1.73 m^2^) [8]. In a long-term study in pigs, an HP diets (35% of total energy consumption, TEC) resulted in enlarged kidneys accompanied by histological damage as well as renal and glomerular volumes being 60–70% higher when compared to control animals (protein intake  = 15% of TEC) [9]. Moreover, risk of kidney stone formation due to high urinary calcium excretion was increased in healthy subjects following an HP dietary protocol for 6 weeks [10]. In contrast to these findings, a 2-year trial in non-diabetic obese individuals reported that an HP diet was not associated with harmful effects on GFR, urinary albumin excretion, or fluid and electrolyte balance compared with a NP diet [11]. However, evidence indicates that obesity itself may accelerate the progression of CKD, induced by pathophysiological mechanism such glomerular hyperfiltration/hypertrophy caused by the raised metabolic needs of the obese individual [12]. In addition, due to the National Health and Nutrition Examination Survey III (NHANES) data, approximately 30% of the US population feature characteristics of reduced kidney function (GFR  = 60–89 ml/min/1.73 m^2^) increasing with age > 40 years [13]. Regarding the increased prevalence of overweight and obesity in this age group, HP diets might thus not represent a reasonable tool in weight management programs even for subjects without an established kidney dysfunction. Therefore, it was the aim of the present systematic review to examine the effects of HP versus LP/NP diets on parameters of renal function in adult subjects. To the best of our knowledge, this is the first meta-analysis performed to investigate the effects of HP diets on outcomes of renal function in subjects without CKD (GFR ≥ 60 ml/min/1.73 m^2^).

## Methods

### Data Sources and Searches

Queries of literature were performed using the electronic databases MEDLINE (until 27^th^ February 2014), EMBASE (until 27^th^ February 2014), and the Cochrane Trial Register (until 27^th^ February 2014) with restrictions to randomized controlled trials, but no restrictions to language and calendar date using the following search term: *("protein")* AND *("renal" OR "kidney" OR "glomerular filtration" OR "creatinine" OR "urea" OR "albumin" OR "calcium")*. Moreover, the reference lists from retrieved articles were checked to search for further relevant studies. This systematic review was planned, conducted, and reported in adherence to standards of quality for reporting meta-analyses [14]. Literature search was conducted independently by both authors, with disagreements resolved by consensus.

### Study Selection

Studies were included in the meta-analysis if they met all of the following criteria: *(i)* randomized controlled or cross-over design; *(ii)* minimum intervention period of 1 week; *(iii)* comparing a HP dietary intervention with a NP/LP intervention (using a 5% difference in total energy intake, as defined previously by Santesso et al. 2012 [1]) that were designed for weight loss or not; *(iv)* age: ≥ 18 years; *(v)* sample size: healthy, overweight, obese, type 2 diabetes (T2D); *(vi)* assessment of the “outcome of interest” markers: GFR, serum creatinine, serum urea, urinary calcium excretion, urinary albumin excretion, serum uric acid, urinary pH; *(vii)* report of post-intervention mean values (if not available, change from baseline values were used) with standard deviation (or basic data to calculate these parameters: standard error, 95% confidence interval, p-values). If data of ongoing studies were published as updates, results of only the longest duration periods were included. Studies enrolling subjects with CKD (GFR <60 ml/min/1.73 m^2^), type 1 diabetes, and macroalbuminuria were excluded. Trials were included if subjects had microalbuminuria, since data from the NHANES III indicated that 12% of the included population suffered from microalbminuria, whereas only 1.5% had macroalbuminuria [15].

### Data Extraction and Quality Assessment

The risk of bias assessment tool by the Cochrane Collaboration was applied specifying the following bias domains: selection bias (random sequence generation, allocation concealment), performance/detection bias (blinding of participants and personnel/blinding of outcome assessment), attrition bias (incomplete data outcome), and reporting bias (selective reporting) [16] (Figure S1).

The following data were extracted from each study: the first author's last name, year of publication, study length, gender distribution and age, BMI, % diabetics, sample size, protein intake (% of total energy content, TEC or g * kg body weight^−1^ * d^−1^), protein origin, calcium intake, energy content of HP and NP/LP diets, outcomes and post mean values or differences in mean of two time point values with corresponding standard deviation.

### Data Synthesis and Analysis

For each outcome measure of interest, a meta-analysis was performed in order to determine the pooled effect of the intervention in terms of weighted mean differences (MDs) between the post-intervention (or change from baseline) values of the HP and NP/LP groups. Combining both the post-intervention values and difference in means in one meta-analysis is an accepted method described by the Cochrane Collaboration [17]. All data were analyzed using the REVIEW MANAGER software provided by the Cochrane Collaboration (http://ims.cochrane.org/revman). The random-effects model was used to estimate MDs with 95% confidence intervals (CIs). Forest plots were generated to illustrate the study-specific effect sizes along with a 95% CI. Heterogeneity between trial results was tested with a standard χ^2^ test. The I^2^ parameter was used to quantify any inconsistency: I^2^ = [(Q – d.f.)]/Q×100%, where Q is the χ^2^ statistic and d.f. is its degrees of freedom. A value for I^2^ greater than ≥ 75% was considered to indicate considerable heterogeneity [18]. Funnel plots were sketched to indicate potential publication bias (e.g. the tendency for studies yielding statistically significant results to be more likely to be submitted and accepted for publication). To evaluate substantial heterogeneity, several post hoc univariate random-effects meta-regressions were performed to examine the association between age, BMI, study length, and % protein intake as independent variables, and changes in GFR, creatinine, urea, and pH (were substantial heterogeneity could be detected) as depending variables, respectively. The p*-values* for differences in effects between the covariates were obtained using the *metareg* command of Stata 12.0 (Stata-Corp, College Station, TX. USA). Two sided *p-values* <0.05 were considered to be statistically significant. To determine the presence of publication bias, the symmetry of the funnel plots in which mean differences were plotted against their corresponding standard errors were assessed.

## Results

### Literature Search

A total of 30 trials (32 reports) extracted from 15734 articles met the inclusion criteria and were analyzed in the systematic review (References S1). The detailed steps of the meta-analysis article selection process are given as a flow chart in Figure S2. Although in accordance with the overall inclusion criteria, one trial was excluded due to inconsistencies in the mean GFR (≤60 ml/min/1.73 m^2^ in 45% of the study population), which was considered to increase the potential for selection bias (Figure S1) [19].

### Characteristics of Studies and Participants

All studies included in this systematic review were RCTs with a duration ranging between 1 week and 24 months, published between 1993 and 2013, and enrolling a total of 2160 participants. All studies compared a HP diet to a NP/LP regimen. The mean age of participants varied between 22.3 and 67 years. Protein intakes in the HP groups were mostly of animal origin except for one trial, where wheat gluten protein was used [20]. General study characteristics are given in Table 1.

**Table 1:** Characteristics of the included studies in the meta-analyses.

References (References S1)	Sample size	Mean age (yrs),	Duration (weeks)	Study design	Protein (g * kg body weight^−1^ * d^−1^, % of TEC)	Protein sources in the HP group	Calcium (mg/d)	Daily energy (kcal)	Microalbuminuria	GFR- measurement
	Mean baseline BMI (kg/m^2^)	Female, %								
	% diabetics									
**Brinkworth et al. 2004**	58	50.2	68	RCT	HP: 30%	30 g skim milk powder, 60 g low-fat cheese, 200 g diet yogurt, 200 g lean meat or poultry, 250 ml low-fat milk	n.d	12-week energy restricted		According to [46], [47] (ml/min)
	34	78%			NP/LP: 15%		n.d	12-week energy restricted		
	0%									
**Brinkworth et al. 2010**	68	51.5	52	RCT	HP: 35%	125 ml milk, 70 g cheddar cheese, 1 egg, 300 g (raw weight) beef, chicken or fish, 100 g (cooked) ham, tuna, beef, turkey, chicken, 40 g raw unsalted nuts	n.d	1433–1672		Modification in renal disease study equation was used [48] (ml/min/1.73 m^2^)
	33.5	64%			NP/LP: 24%		n.d	1433–1672		
	0%									
**Cao et al. 2011**	16	56.9	7	Crossover	HP: 1.6 g; 20%	500 ml milk, 80 g ham, 120 g roast beef, 120 g baked chicken, 50 g steamed peas	865	Isocaloric		
	26.8	100%			NP/LP: 0.8 g; 10%		907	Isocaloric		
	0%									
**Ferrara et al. 2006**	15	26.4	24	RCT	HP: 1.9 g	Beaf, pork, ham, poultry	n.d	n.d		
	23.5	0%			NP/LP: 1.3 g		n.d	n.d		
	0%									
**Frank et al. 2009**	24	24.1	1	crossover	HP: 2.4 g; 26.6%; 21.7% (animal protein)	Animal sources including milk and milk products	n.d	2743		Assessed on the basis of sinistrin clearence (ml/min)
	22.3	0%			NP/LP: 1.2 g; 13.3%; 7.4% (animal protein)		n.d	2736		
	0%									
**Friedman et al. 2012**	307	45.5	104	RCT	HP: LC diet	Unlimited protein cosnumption	n.d	n.d		Calculated by dividing the 24-hr urinary creatinine excretion (mg/d) by 1440 (min/d) and then dividing aigan by the serum creatinine (mg/dl) x100. (ml/min)
	36.1	68%			NP/LP: 15%		n.d	1200–1500		
	0%									
**Gross et al. 2002**	28	57.3	4	Crossover	HP: 1.4 g	Chicken	712	n.d	x	Measured using the ^51^ Cr-EDTA single-injection technique (ml/min/1.73 m^2^)
	26.3	25%			NP/LP: 0.66 g		732	n.d		
	100%									
**Jenkins et al. 2001**	20	55.6	4	Crossover	HP: 27.4%; 20.1% (vegetable protein)	80 g wheat gluten protein (bread)	n.d	2764		Not described (ml/min)
	26	25%			NP/LP: 15.6%; 8.2% (vegetable protein)		n.d	2835		
	0%									
**Jesudason et al. 2013**	45	60.9	12	RCT	HP: 30%	90–120 g/d	n.d	1435–1674	x	Calculated from serum creatinine using the Modification of diet and renal disease study formula [49](ml/min/1.73 m^2^)
	36	22%			NP/LP: 20%		n.d	1435–1674		
	100%									
**Johnston et al. 2004**	20	19–54	6	RCT	HP: 31.5%	Egg beater scramble with 28 g ham and 28 g cheese, 1 l milk, 84 open faced turkey, 28 g provolone, chicken chow mien dinner, peas and beans	1828	1700		Not described (ml/s/m^2^)
	28.9	90%			NP/LP: 15%		1187	1700		
	0%									
**Juraschek et al. 2013**	164	53.5	6	Cross-over	HP: 25%, 12.5%	Food sources used for protein replacement primarily were vegetable-based	n.d	n.d		eGFR was calculated using the CKD Epidemiology Collaboration cystatin C equation [43] (ml/min/1.73 m^2^)
	30.2	45%			NP/LP: 15%, 5.4%		n.d	n.d		
	0%									
**Krebs et al. 2012**	419	57.8	104	RCT	HP: 30%	n.d	n.d	−500	x	
	36.6	60%			NP/LP: 15%		n.d	−500		
	100%									
**Larsen et al. 2011**	99	59.4	52	RCT	HP: 30%	A combination of lean meat, chicken and fish	n.d	12-week energy restricted	x	Not described (ml/min/1.73 m^2^)
	27–40	52%			LP/NP: 15%		n.d	12-week energy restricted		
	100%									
**Leidy et al. 2007**	46	50	12	RCT	HP: 1.4 g; 30%	180 g cooked pork, loin, ham, or Canadian bacon	n.d	−750		Calculated from serum creatinine using the Modification of diet and renal disease study formula [49] (ml/min/1.73 m^2^)
	30.6	100%			NP/LP: 0.8 g; 18%		n.d	−750		
	0%									
**Li et al. 2010**	100	49.3	52	RCT	HP: 2.2 g, 30%	Formula 1, Herbalife Intl., Los Angeles	n.d	−500		Not described (ml/min)
	34.5	66%			NP/LP: 1.1 g, 15%		n.d	−500		
	0%									
**Liu et al. 2013**	50	47.9	12	RCT	HP: LC diet	Boiled eggs 2 (ad libitum for snacks, Lactalbumin (15 g), Duck leg (220 g)	n.d	ad libitum		
	26.7	100%			NP/LP: 18%		n.d	1500		
	0%									
**Luger et al. 2013**	44	62.4	12	RCT	HP: 30%	Received data sheets referring to protein-rich foods: major high-protein sources included: soy-based foods, milk products, fish and poultry	n.d	1272	x	Calculated from serum creatinine using the Modification of diet and renal disease study formula [49](ml/min/1.73 m^2^)
	33.3	55%			NP/LP: 15%		n.d	1235		
	100%									
**Luscombe-Marsh et al. 2005**	73	20–65	16	RCT	HP: 40%	400 ml skim milk, 40 g skim milk powder, 40 g low-fat cheese, 300 g meat, poultry or fish, 20 g almonds, 200 g low-fat artificially sweetened yogurt	n.d	12-week energy restricted		(urine creatinine concentration in mmol/l x urine volume in ml/1140 min/ plasma creatinine concentration in µmol/l×1000 ml x min)×0.7. (ml/min)
	27–40	65%			NP/LP: 20%		n.d	12-week energy restricted		
	0%									
**Noakes et al. 2005**	100	49.5	12	RCT	HP: 34%	250 ml milk, 200 g low-fat yogurt, 300 g lean meat, poultry or fish	777	1342		Not described (ml/min)
	32.5	100%			NP/LP: 17%		594	134		
	0%									
**Nuttall et al. 2003;Gannon et al. 2003**	12	n.d	5	Crossover	HP: 30%	1 l low-fat milk, 113 g beef, 255 g baked chicken, 113 g low-fat cheese, 227 g low-fat yogurt	n.d	2235	x	
	n.d	17%			NP/LP: 15%		n.d	2266		
	100%									
**Nuttall et al. 2006**	8	63	5	Crossover	HP: 30%	124 g egg substitute, 56 g cheddar cheese, 226 g roasted ham, 85 g swiss cheese, 253 split pea soup, 170 g tuna, 80 g peas, 56 g dry-roasted peanuts	n.d	Isocaloric	x	
	31	0%			NP/LP: 15%		n.d	Isocaloric		
	100%									
**Pomerleau et al. 1993**	20	58	3	Crossover	HP: 1.9 g; 22%;	Supplements (casein, gelatin, vegetable proteins, yeast, and soy)	883	2182	x	^99m^Technicum-DTPA (diethylenetriamine pentaacetic acid) plasma clearance (ml/s/1.73 m^2^)
	33	33%			NP/LP: 0.8 g; 10%		930	2110		
	100%									
**Roughead et al. 2003**	15	60.5	8	Crossover	HP: 1.62 g; 20%	Pork, turkey breast, beef round, ham, chicken breast	596	2296		Calculated from serum and urinary creatinine, which were measured using alkaline picric acid (ml/s)
	26.5	100%			NP/LP: 0.94 g; 12%		617	2296		
	0%									
**Sargrad et al. 2005**	12	47.5	8	RCT	HP: 30%	Chicken, fish, eggs, low-fat milk, cheeses, nuts	567	1275	n.d	
	34.5	75%			NP/LP: 15%		521	1371		
	100%									
**Skov et al. 1999**	50	39.6	24	RCT	HP: 25%	Dairy products and meat, the latter represented by both beef, pork, poultry, lamb	n.d	ad libitum		Measured using the ^51^Cr-EDTA single-injection technique (ml/min)
	30.4	76%			NP/LP: 12%		n.d	ad libitum		
	0%									
**Stern et al. 2004**	132	53.5	12	RCT	HP: LC diet	Unlimited: meat, fowl, fish, shellfish, eggs, 110 g hard cheese	n.d	−500		
	42.9	17%			NP/LP: 15%		n.d	no		
	41%									
**Velázquez Lopez et al. 2008**	41	67	4	RCT	HP: 1–1.2 g	n.d	n.d	isocaloric	x	Assessed using creatinine-clearance estimation by the Cockroft and Gault formula [50] (ml/min)
	26.82	65%			NP/LP: 0.6–0.8 g		n.d	Isocaloric		
	100%									
**Wagner et al. 2007**	22	30.8–60.2	1	Crossover	HP: 2 g	Meat, dairy products, and egg white powder	n.d	Isocaloric		Calculated from serum creatinine using the Modification of diet and renal disease study formula [49] (ml/min/1.73 m^2^)
	25.5	69%			NP/LP: 0.5 g		n.d	Isocaloric		
	0%									
**Westman et al. 2008; Yancy et al. 2007**	84	51.8	24	RCT	HP: VLC diet	Unlimited: meat, fowl, fish, shellfish, eggs, 120 g hard cheese; 60 g fresh cheese	n.d	ad libitum	x	
	38	78%			NP/LP: 15%		n.d	−500		
	100%									
**Wycherley et al. 2012**	68	50.8	52	RCT	HP: 35%	3 serves low-fat dairy, 300 g lean red meat, 100 deli-scliced meat/canned fish	n.d	1680		According to [51] (ml/min/1.73 m^2^)
	33	0%			LP: 17%		n.d	1680		
	0%									

BMI, Body-Mass-Index; CKD, chronic kidney disease; HP, high protein; n.d, no data; NP/LP, normal/low protein; RCT, randomized controlled trial; VLC, very-low carbohydrate.

### Outcomes

The pooled estimates of effect size for the effects of HP as compared to NP/LP on outcomes of kidney function are summarized in Table 2. Changes in serum creatinine (Figure S3), urinary albumin excretion (Figure S5), uric acid (Figure S4), and urinary pH (Figure S7) were not significantly different following HP diets as compared to NP/LP diets and are given as Supplementary material.

**Table 2:** Pooled estimates of effect size (95% confidence intervals) expressed as weighted mean difference for the effects of HP vs. NP/LP diets on outcomes of renal function.

Outcomes	No. of Studies	Sample size	MD	95% CI	p-values	Inconsistency I^2^
GFR (ml/min/1.73 m^2^)	21	1599	7.18	[4.45, 9.91]	<0.001	52%
Creatinine (µmol/l)	22	1764	−1.42	[−3.50, 0.65]	0.18	57%
Urea (mmol/l)	13	910	1.75	[1.13, 2.37]	<0.001	88%
Uric acid (µmol/l)	8	295	0.18	[−0.08, 0.44]	0.17	3%
Urinary pH	7	210	−0.39	[−0.82, 0.03]	0.07	95%
Urinary Albumin/Protein (mg/24h)	11	783	0.50	[−2.83, 3.82]	0.77	63%
Urinary calcium excretion (mg/24h)	10	708	25.43	[13.62, 37.24]	<0.001	90%

**Figure 1:**
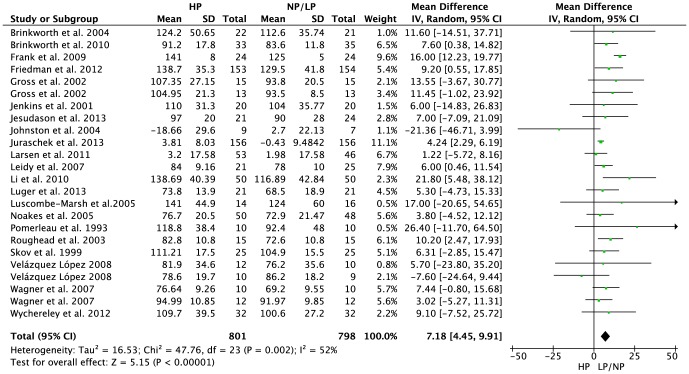
Forest plot showing pooled MD with 95% CI for glomerular filtration rate (ml/min/1.73 m^2^) of 21 randomized controlled HP diet trails. For each high protein study, the shaded square represents the point estimate of the intervention effect. The horizontal line joins the lower and upper limits of the 95% CI of these effects. The area of the shaded square reflects the relative weight of the study in the respective meta-analysis. The diamond at the bottom of the graph represents the pooled MD with the 95% CI. HP, high protein; NP/LP, normal protein/low protein.

**Figure 2:**
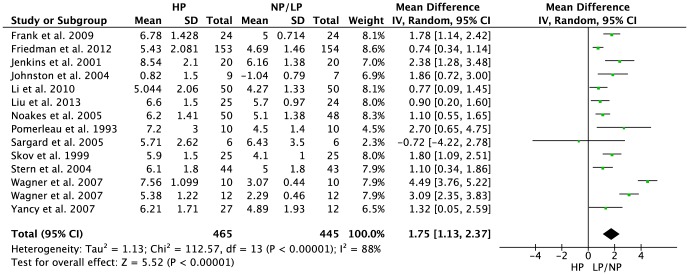
Forest plot showing pooled MD with 95% CI for serum urea (mmol/l) of 13 randomized controlled HP diet trails. For each high protein study, the shaded square represents the point estimate of the intervention effect. The horizontal line joins the lower and upper limits of the 95% CI of these effects. The area of the shaded square reflects the relative weight of the study in the respective meta-analysis. The diamond at the bottom of the graph represents the pooled MD with the 95% CI. HP, high protein; NP/LP, normal protein/low protein.

HP diets were associated with a significantly more pronounced increase in GFR as compared to NP/LP protocols [MD: 7.18 ml/min/1.73 m^2^ (95% CI 4.45 to 9.91), p<0.001] (Figure 1). Serum urea [MD: 1.75 mmol/l (95% CI 1.13 to 2.37), p<0.001] (Figure 2) and urinary calcium excretion [MD: 25.43 mg/24h (95% CI 13.62 to 37.24), p<0.001] (Figure S6) were significantly more increased by HP diets in comparison to the NP/LP settings, respectively.

### Sensitivity Analyses

Including RCTs investigating only subjects without T2D (18 trials) confirmed the results or the primary meta-analysis (Table S1). Furthermore, with the exception of urinary pH (only 1 trial) and urinary calcium excretion (5 trials), the main results could be confirmed when including only obese subjects (20 trials) (Table S2). Similar observations could be made for long-term trials (≥ 12 weeks, 17 trials) (Table S3). Sensitivity analysis including only T2D subjects resulted in similar observations (Table S4). Following exclusion of the trial by Jenkins et al. [20] (being the only RCT not using animal protein as a source for supplementations in HP protocols), HP diets resulted in a significantly more pronounced increase in uric acid as compared to NP/LP protocols.

### Publication Bias

The funnel plots (with respect to effect size changes for markers of kidney health in response to HP diets, respectively) indicates little to moderate asymmetry, suggesting that publication bias cannot be completely excluded as a factor of influence on the present meta-analysis (Figure S8-S14). It remains possible that small studies yielding inconclusive data have not been published or failed to do so.

### Heterogeneity

Considerable heterogeneity was found with respect to serum urea (I^2^ = 88%), urinary pH (I^2^ = 95%), and urinary calcium excretion (I^2^ = 90%) (Table 2). It was assumed that high heterogeneity might be explained by non-uniform study characteristics in the high protein groups such as variations in age, BMI, study length, and protein intake. To gain insight into these potential correlations, a random-effects meta-regression was performed to examine the associations between HP and NP/LP group parameters and changes in GFR, serum creatinine, serum urea, uric acid, urinary albumin, and urinary pH, respectively. A statistically significant dose-response relationship could be detected between protein intake and increases in serum urea (p = 0.023) No such correlations could be detected between the other study characteristics and parameters mentioned.

## Discussion

It was the aim of the present meta-analysis to investigate the impact of HP vs. LP/NP diets on parameters of kidney function in subjects without an established CKD. The main findings suggest that subjects following an HP diet presented themselves with increased GFR, serum urea, and urinary calcium excretion, respectively. Further increases in serum concentrations of uric acid could be observed in those individuals following an HP regimen, when the trial by Jenkins et al. [20] was excluded from the analysis due to the fact, that it was the only study using vegetable protein exclusively as a supplement.

The choice of diet as a tool in weight management often includes variations in macronutrient composition differing from the regular recommendations of national as well as international authorities. Due to their proposed effects on thermogenesis and satiety, HP diets have gained increasing interest in recent years. The potential detrimental effects of HP diets on kidney function are still discussed controversially. In a long-term study in rats, feeding an HP diet (35% of TEC) resulted in a significant reduction in body weight, however this was accompanied by 17% higher kidney weights, a 3-fold raise in proteinuria, larger glomeruli, and a 27% increase in creatinine clearance as compared to the NP (15% of TEC) feed rats, respectively [21]. Other detrimental effects of HP diets on kidney function include higher organ weight, and histologically detectable tissue damage [9]. Despite the limited transferability of results gained in animal experiments, pathophysiological side-effects of HP diets could appear in humans as well. At least in patients with established CKD, reducing protein intake decreases the occurrence of renal death by 32% when compared to higher/unrestricted protein intake [22]. Data of a meta-analysis investigating 17 cohort studies suggest that HP/lower carbohydrate intakes were associated with increased all-cause mortality [23].

Some 30 years ago, Brenner et al. [24] expressed the hypothesis that an increase in GFR and glomerular pressure might cause renal dysfunction and raise the risk for renal injury. Although this hypothesis could neither be validated nor refuted to date, one might argue that long-term HP intakes exert harmful effects on kidney function by causing renal hyperfiltration. Concerning the mechanism mediating the increased GFR, Frank et al. [25] hypothesized that protein load induces a vasodilatatory response leading to hyperemia. In a meta-analysis of 14 observational studies enrolling 105.872 participants, a GFR > 105 ml/min/1.73 m^2^ was associated with an increased risk of all-cause mortality [26]. However, the authors of this study stated that their findings should be interpreted conservatively. Instead of being a pathophysiological reaction, HP-induced changes in kidney function such as the increase in GFR might as well represent a physiological adaptation process [27], [28]. The capacity of the kidney to increase functional level with protein intake suggest a renal function reserve [29].

The raise in serum uric acid concentrations observed in the present meta-analysis in individuals following an HP diet was most likely probably caused by the higher intake of animal source foods rich in purines. Epidemiological data suggest that protein per se does not raise serum uric acid [30]. Among others, the Health Professionals Follow-up Study observed a 41% increase in the risk of first attack of gout when comparing the highest vs. lowest meat consumption quintile [31]. In addition to gout disorders, serum uric acid has been described as a modifiable risk factor for CVD and all-cause mortality in men and women [32], [33]. From these data, one may conclude that the source of protein is of higher importance than its absolute amount. A 26-year follow up of the Nurses' Health Study (NHS) revealed that protein sources such as red meat and high-fat dairy products were significantly associated with an elevated risk of CHD, while higher intakes of poultry, fish, and nuts (although rich in protein as well) were correlated with a lower risk of CHD [34]. In contrast to these findings, Bernstein et al. [35] concluded that long-term consumption of high-protein diets may cause renal injury and accelerate the onset of CKD in persons with normal renal function independent of the fact, whether the protein food source is either predominantly animal or vegetable protein.

Reductions in urinary pH (p =  0.07) as observed in this meta-analysis for HP diets are regarded as an independent risk factor for nephrolithiasis [7]. In addition, HP intake raised urinary calcium excretion which is a common characteristic in patients with calcareous stones [36], [37]. Impairment of calcium homeostasis might lead to a decrease in bone mineral density. However, clinical and epidemiological data do not support the concept that HP diets exert harmful effects on bone health.[1], [38] Moreover, the differences observed in the present meta-analysis do not seem to be clinically relevant.

Two meta-analyses including observational studies showed that overweight, obesity and the metabolic syndrome increase the risk of kidney disease by 40 to 83% [39], [40]. Considering that some two thirds the trials included in the present meta-analysis were enrolling obese subjects, it could be speculated that a high protein intake will add another detrimental factor to the increased risk of kidney dysfunction already established for this population. According to the recommendations of the American Diabetes Association, patients with T2D should not refer to HP diets as a means for weight loss due to the unknown long-term effects of protein intakes > 20% of TEC [41].

### Limitations

Regarding the validity of the main outcome parameter GFR, the creatinine-based estimating equations used in the trials included in this systematic review are known to have some limitations with respect to precision as well as being affected by variations in protein intake, which might be further aggravated by the fact that the study population did not suffer from manifested chronic kidney disease. Thus, the GFR effects observed in the present meta-analysis have to be interpreted in a conservative manner, since increased creatinine values would translate into a lower estimated GFR [42]. A cross-sectional study by Inker et al. has shown that cystatin C might represent a more useful marker for estimating GFR especially when combined with creatinine [43]. Moreover, a post hoc analysis of the “Modification of Diet and Renal Disease” study (the origin of eGFR based on serum creatinine) has shown that dietary protein reduced the change in creatinine, but did not significantly affect cystatin C changes [44].

Other limitations of the present review include the limited number of studies and the heterogeneity of the study designs. Thus, this meta-analysis does not consider unpublished data. Examination of funnel plots showed little to moderate asymmetry suggesting that publication bias cannot be completely excluded as a confounder of the present meta-analysis (e.g. lack of published studies with inconclusive results) which may have had at least a moderate impact on the effect size estimates. A major limitation of nutritional intervention trials is the heterogeneity of various aspects and characteristics of the study protocols. Therefore, it is not surprising that the RCTs and crossover studies included in the present analyses varied regarding type(s) of diets used (energy restriction, isocaloric), definitions of HP and NP/LP diets, study population (i.e. age, sex, healthy, overweight, obese, type 2 diabetics), intervention time (1–108 weeks), as well as nutritional assessment. Following sensitivity analyses excluding only trials enrolling patients with T2D, the effect of HP diets on GFR remained the same as those observed in the conclusive analyses. With respect to other potential modulating variables, sensitivity analyses and meta-regressions failed to show any correlations between the findings of the meta-analyses and age, gender, BMI, and study duration and % protein intakes (data not shown). Only few studies provided information on the quality of their respective setup (e.g. method of randomization, follow-up protocol with reasons for withdrawal, see Figure S1 for Risk of bias assessment according to the Cochrane Collaboration) demanding a conservative interpretation of results. To estimate GFR heterogeneous equation were used (see Table 2). Moreover, the included trials varied with respect to dietary assessment methods to validate adherence of participants. In an HP dietary intervention study by Friedman et al. [11], significant increases in serum creatinine clearance were found after 3 and 12 months, but were not detectable anymore following a 24 month interval, indicating that adherence to the HP diet was not present at the end of the trial. In addition, Krebs et al. [45] could not measure significant differences in renal function at any time-point (6, 12, and 24 months) when comparing an HP with a LP regimen. Assessment of protein intakes revealed that the difference between the two groups did not exceed more than 2% of TEC suggesting a very low adherence to the dietary interventions. Therefore, adherence of individuals assigned to a HP diet might change over time. Although adherence is usually good in the short term, the long-term effects of HP vs. LP/NP diets are of higher interest. Augmentations of urinary calcium excretions found in the present meta-analysis in individuals following an HP diet might be interpreted as an adherence marker of HP diets. Some of the present meta-analyses were done using both post-intervention values and changes in mean difference, however, this was considered to be an acceptable procedure as described by the Cochrane Collaboration [17]. On the other hand, this meta-analysis has several strengths as well. All analyses were conducted following a stringent protocol, e.g. participants were randomly assigned to the intervention groups in all trials. Randomized controlled trials are considered to be the gold standard for evaluating the effects of an intervention and are subject to fewer biases as compared to observational studies. With a sample size of 2160 volunteers, the present meta-analysis provides the power to detect statistically significant mean differences as well as to assess publication bias.

In conclusion, HP diets were associated with increased GFR, serum urea, urinary calcium excretion, and serum concentrations of uric acid. Most of these changes could be interpreted as physiological adaptive mechanism induced by HP diet without any clinical relevance. However, considering of the fact that subclinical CKD is highly prevalent, and that obesity is associated with kidney disease, weight reduction programs recommending HP diets especially from animal sources should be handled with caution.

## Supporting Information

Figure S1 **Risk of bias assessment tool.**
(EPS)

Figure S2 **Flow chart.**
(DOCX)

Figure S3 **Forest plot showing pooled MD with 95% CI for serum creatinine.**
(EPS)

Figure S4 **Forest plot showing pooled MD with 95% CI for serum uric acid.**
(EPS)

Figure S5 **Forest plot showing pooled MD with 95% CI for urinary albumin/protein excretion.**
(EPS)

Figure S6 **Forest plot showing pooled MD with 95% CI for urinary calcium excretion.**
(EPS)

Figure S7 **Forest plot showing pooled MD with 95% CI for urinary pH.**
(EPS)

Figure S8 **Funnel plot: glomerular filtration rate.**
(EPS)

Figure S9 **Funnel plot: serum creatinine.**
(EPS)

Figure S10 **Funnel plot: serum urea.**
(EPS)

Figure S11 **Funnel plot: serum uric acid.**
(EPS)

Figure S12 **Funnel plot: urinary pH.**
(EPS)

Figure S13 **Funnel plot: urinary albumin/protein excretion.**
(EPS)

Figure S14 **Funnel plot: urinary calcium excretion.**
(EPS)

Table S1 **Sensitivity analysis for subjects without T2D.**
(DOCX)

Table S2 **Sensitivity analysis for obese subjects.**
(DOCX)

Table S3 **Sensitivity analysis for long-term studies (≥12 weeks).**
(DOCX)

Table S4 **Sensitivity analysis for T2D subjects.**
(DOCX)

Checklist S1 **PRISMA checklist.**
(DOCX)

References S1 (DOCX)
