# Supplementary material for: Comparison of High vs. Normal/Low Protein Diets on Renal Function in Subjects without Chronic Kidney Disease: A Systematic Review and Meta-Analysis
Source: PLoS One. 2014 May 22;9(5):e97656. doi: 10.1371/journal.pone.0097656 (PMC4031217; doi:10.1371/journal.pone.0097656)
Supplement: Figure S2 — Flow chart. (DOCX) [file pone.0097656.s002.docx]

Records identified through database searching: (until 27th February 2014)
PUBMED (n=5942)

Cochrane Library (n=6835)

EMBASE (n=2955)

Additional records identified through other sources
(n =2)

Records screened
(n =15734)

Records excluded: title, abstract, not relevant, case-report, cross-sectional study, review, mechanism study

(n=15614)

Full-text articles assessed for eligibility
(n =120)

Full-text articles excluded, with reasons (n =90)

Macroalbuminuria (n=36)

Chronic kidney disease (n=43)

No data (n=7)

Lack of a comparison (n=4)

Studies included in qualitative synthesis
(n = 30)

Studies included in quantitative synthesis (meta-analysis)

n=30 (32 reports)
