# Supplementary material for: Comparison of High vs. Normal/Low Protein Diets on Renal Function in Subjects without Chronic Kidney Disease: A Systematic Review and Meta-Analysis
Source: PLoS One. 2014 May 22;9(5):e97656. doi: 10.1371/journal.pone.0097656 (PMC4031217; doi:10.1371/journal.pone.0097656)
Supplement: Table S1 — Sensitivity analysis for subjects without T2D. (DOCX) [file pone.0097656.s015.docx]

| **Outcomes** | **No. of**  **Studies** | **Sample size** | **MD** | **95% CI** | **p-values** | **Inconsistency I^2^** |
| --- | --- | --- | --- | --- | --- | --- |
| GFR (ml/min/1.73m^2^) | 15 | 1296 | 7.73 | [4.43, 11.04] | <0.001 | 63% |
| Creatinine (µmol/l) | 14 | 1205 | -2.64 | [-4.92, -0.35] | 0.02 | 61% |
| Urea (mmol/l) | 9 | 752 | 1.87 | [1.13, 2.61] | <0.001 | 92% |
| Uric acid (µmol/l) | 5 | 168 | 0.14 | [-0.26, 0.55] | 0.49 | 43% |
| Urinary pH | 5 | 170 | -0.46 | [-0.99, 0.08] | 0.09 | 96% |
| Urinary Albumin/protein (mg/24h) | 4 | 501 | 2.92 | [-3.23, 9.08] | 0.35 | 85% |
| Urinary calcium excretion (mg/24h) | 6 | 529 | 27.34 | [14.30, 40.38] | <0.001 | 94% |
