# Supplementary material for: Comparison of High vs. Normal/Low Protein Diets on Renal Function in Subjects without Chronic Kidney Disease: A Systematic Review and Meta-Analysis
Source: PLoS One. 2014 May 22;9(5):e97656. doi: 10.1371/journal.pone.0097656 (PMC4031217; doi:10.1371/journal.pone.0097656)
Supplement: Table S2 — Sensitivity analysis for obese subjects. (DOCX) [file pone.0097656.s016.docx]

| **Outcomes** | **No. of**  **Studies** | **Sample size** | **MD** | **95% CI** | **p-values** | **Inconsistency I^2^** |
| --- | --- | --- | --- | --- | --- | --- |
| GFR (ml/min/1.73m^2^) | 14 | 1324 | 4.96 | [3.38, 6.54] | <0.001 | 0% |
| Creatinine (µmol/l) | 16 | 1561 | -1.35 | [-4.22, 1.51] | 0.35 | 69% |
| Urea (mmol/l) | 9 | 762 | 1.06 | [0.76, 1.36] | <0.001 | 27% |
| Uric acid (µmol/l) | 3 | 152 | 0.19 | [-0.20, 0.57] | 0.35 | 0% |
| Urinary Albumin/protein (mg/24h) | 8 | 674 | -0.22 | [-1.73, 1.29] | 0.78 | 0% |
| Urinary calcium excretion (mg/24h) | 5 | 562 | 46.59 | [-11.26, 104.44] | 0.11 | 88% |
