# Supplementary material for: Comparison of High vs. Normal/Low Protein Diets on Renal Function in Subjects without Chronic Kidney Disease: A Systematic Review and Meta-Analysis
Source: PLoS One. 2014 May 22;9(5):e97656. doi: 10.1371/journal.pone.0097656 (PMC4031217; doi:10.1371/journal.pone.0097656)
Supplement: Table S3 — Sensitivity analysis for long-term studies (≥12 weeks). (DOCX) [file pone.0097656.s017.docx]

| **Outcomes** | **No. of**  **Studies** | **Sample size** | **MD** | **95% CI** | **p-values** | **Inconsistency I^2^** |
| --- | --- | --- | --- | --- | --- | --- |
| GFR (ml/min/1.73m^2^) | 12 | 992 | 6.23 | [3.52, 8.93] | <0.001 | 0% |
| Creatinine (µmol/l) | 14 | 1236 | -1.68 | [-5.14, 1.77] | 0.34 | 67% |
| Urea (mmol/l) | 7 | 730 | 1.03 | [0.75, 1.30] | <0.00001 | 20% |
| Uric acid (µmol/l) | 3 | 151 | 0.18 | [-0.22, 0.58] | 0.37 | 0% |
| Urinary Albumin/protein (mg/24h) | 6 | 618 | -0.04 | [-1.68, 1.60] | 0.96 | 0% |
| Urinary calcium excretion (mg/24h) | 4 | 546 | 50.69 | [-10.49, 111.88] | 0.10 | 91% |
