# Supplementary material for: Comparison of High vs. Normal/Low Protein Diets on Renal Function in Subjects without Chronic Kidney Disease: A Systematic Review and Meta-Analysis
Source: PLoS One. 2014 May 22;9(5):e97656. doi: 10.1371/journal.pone.0097656 (PMC4031217; doi:10.1371/journal.pone.0097656)
Supplement: Table S4 — Sensitivity analysis for T2D subjects. (DOCX) [file pone.0097656.s018.docx]

**Supplementary material**

**
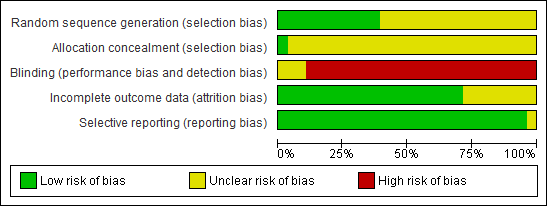
**

Figure S1: Risk of bias assessment tool. Across trials, information is either from trials at a low risk of bias (green), or from trials at unclear risk of bias (yellow), or from trials at high risk of bias (red).

Figure S2: Flow chart for meta-analysis article selection process.

Records identified through database searching: (until 27th February 2014)
PUBMED (n=5942)

Cochrane Library (n=6835)

EMBASE (n=2955)

Additional records identified through other sources
(n =2)

Records screened
(n =15734)

Records excluded: title, abstract, not relevant, case-report, cross-sectional study, review, mechanism study

(n=15614)

Full-text articles assessed for eligibility
(n =120)

Full-text articles excluded, with reasons (n =90)

Macroalbuminuria (n=36)

Chronic kidney disease (n=43)

No data (n=7)

Lack of a comparison (n=4)

Studies included in qualitative synthesis
(n = 30)

Studies included in quantitative synthesis (meta-analysis)

n=30 (32 reports)

**Electronic search strategy (specifics as required for MEDLINE: 27.02.2014)**

Limits used: “randomized controlled trials”, “humans”, and “Adult: 19+ years“

("protein") AND ("renal" OR "kidney" OR "glomerular filtration rate" OR "creatinine" OR "urea" OR "albumin" OR "calcium")

"protein"[All Fields] AND ("renal"[All Fields] OR "kidney"[All Fields] OR "glomerular filtration"[All Fields] OR "creatinine"[All Fields] OR "urea"[All Fields] OR "albumin"[All Fields] OR "calcium"[All Fields]) AND (Randomized Controlled Trial[ptyp] AND "humans"[MeSH Terms] AND "adult"[MeSH Terms])


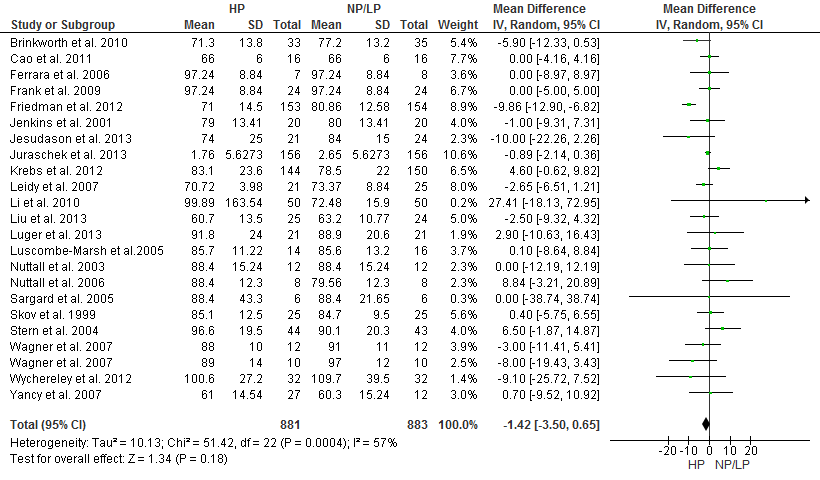
Figure S3. Forest plot showing pooled MD with 95% CI for serum creatinine (µmol/l) for 22 randomized controlled HP diet studies. For each HP study, the shaded square represents the point estimate of the intervention effect. The horizontal line joins the lower and upper limits of the 95% CI of these effects. The area of the shaded square reflects the relative weight of the study in the respective meta-analysis. HP, high protein; NP/LP, normal protein/low protein.


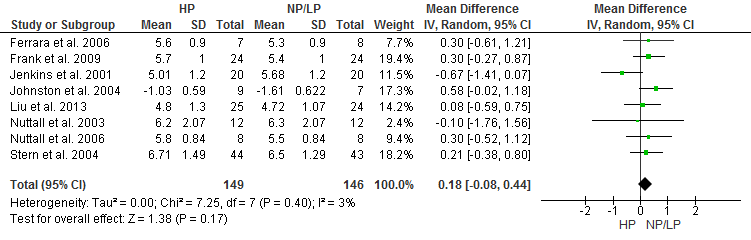
Figure S4. Forest plot showing pooled WMD with 95% CI for serum uric acid (µmol/l) for 8 randomized controlled HP diet studies. For each HP study, the shaded square represents the point estimate of the intervention effect. The horizontal line joins the lower and upper limits of the 95% CI of these effects. The area of the shaded square reflects the relative weight of the study in the respective meta-analysis. HP, high protein; NP/LP, normal protein/low protein.


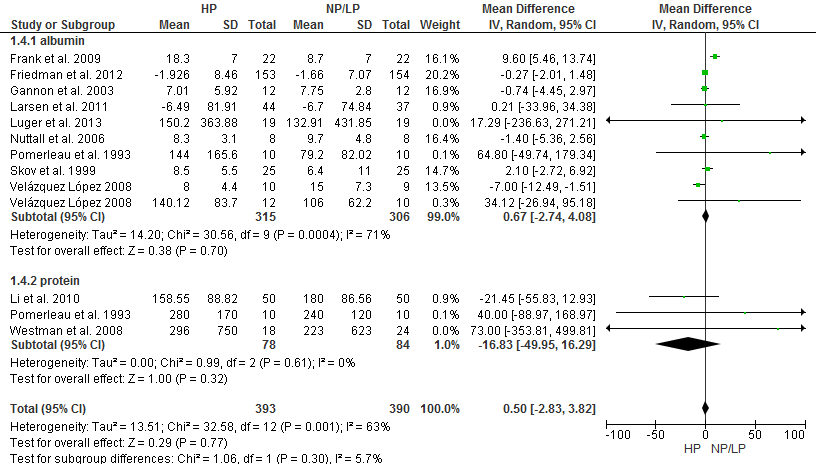
 Figure S5. Forest plot showing pooled MD with 95% CI for urinary albumin/protein excretion (mg/24h) for 11 randomized controlled HP diet studies. For each HP study, the shaded square represents the point estimate of the intervention effect. The horizontal line joins the lower and upper limits of the 95% CI of these effects. The area of the shaded square reflects the relative weight of the study in the respective meta-analysis. HP, high protein; NP/LP, normal protein/low protein.


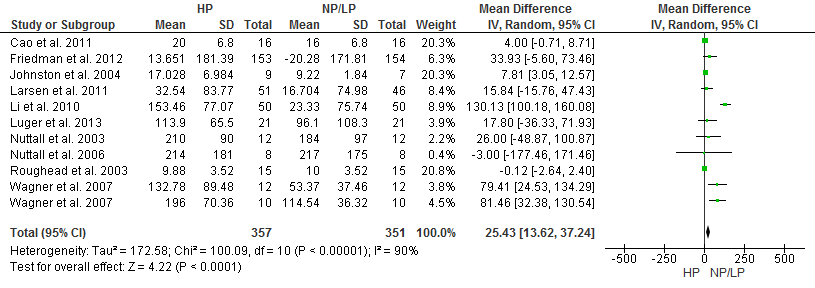


Figure S6. Forest plot showing pooled WMD with 95% CI for urinary calcium excretion (mg/24h) for 10 randomized controlled HP diet studies. For each HP study, the shaded square represents the point estimate of the intervention effect. The horizontal line joins the lower and upper limits of the 95% CI of these effects. The area of the shaded square reflects the relative weight of the study in the respective meta-analysis. HP, high protein; NP/LP, normal protein/low protein.


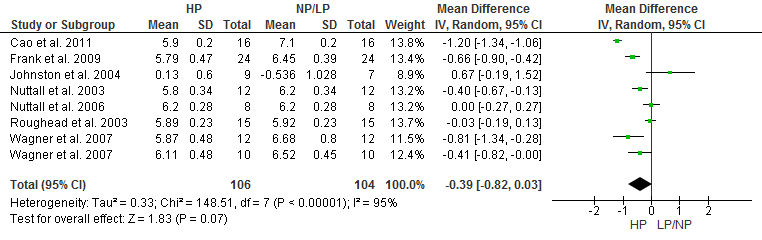
 Figure S7. Forest plot showing pooled MD with 95% CI for urinary pH of 7 randomized controlled HP diet trails.

For each high protein study, the shaded square represents the point estimate of the intervention effect. The horizontal line joins the lower and upper limits of the 95% CI of these effects. The area of the shaded square reflects the relative weight of the study in the respective meta-analysis. The diamond at the bottom of the graph represents the pooled MD with the 95% CI. HP, high protein; NP/LP, normal protein/low protein.


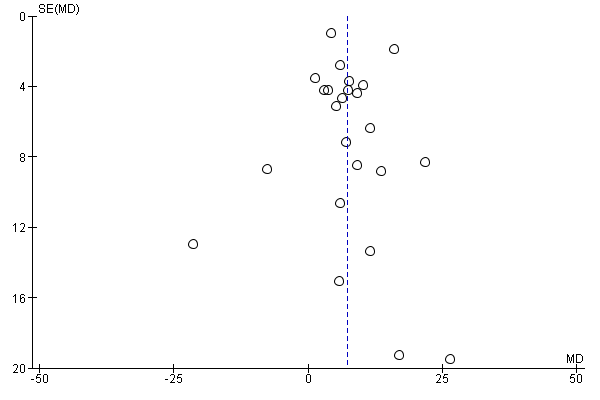


Figure S8. Funnel plot showing study precision against the MD effect estimate with 95% CIs for glomerular filtration rate. SE = Standard error.


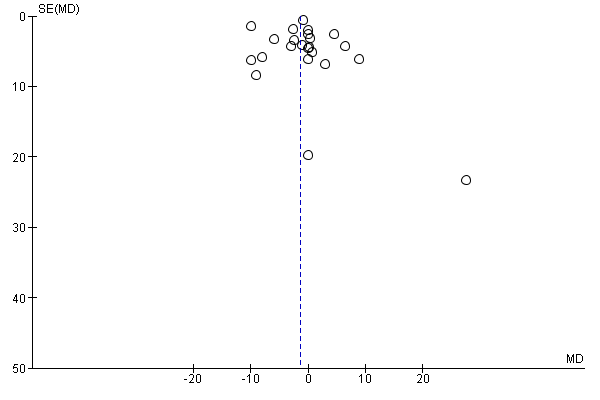
Figure S9. Funnel plot showing study precision against the MMD effect estimate with 95% CI for serum creatinine. SE = Standard error.


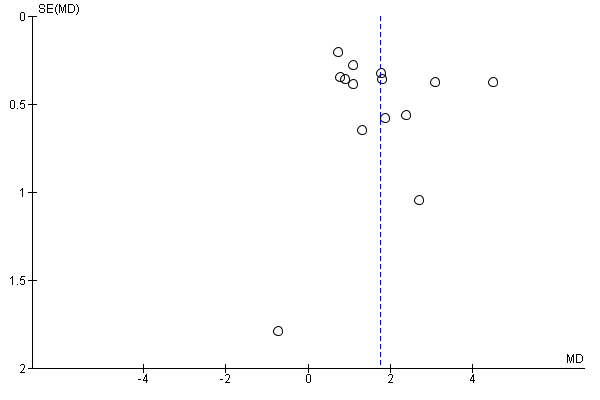


Figure S10. Funnel plot showing study precision against the MD effect estimate with 95% CIs for serum urea. SE = Standard error.


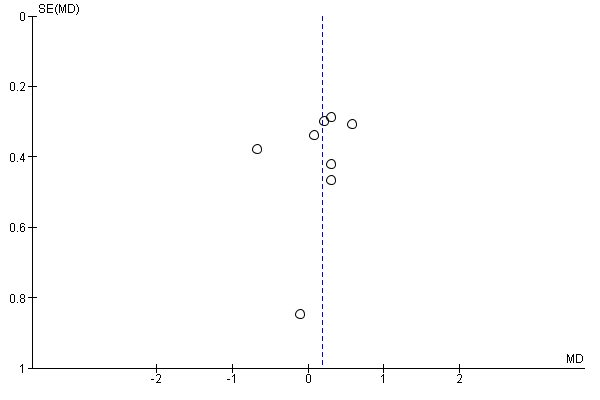


Figure S11. Funnel plot showing study precision against the MD effect estimate with 95% CIs for serum uric acid. SE = Standard error.


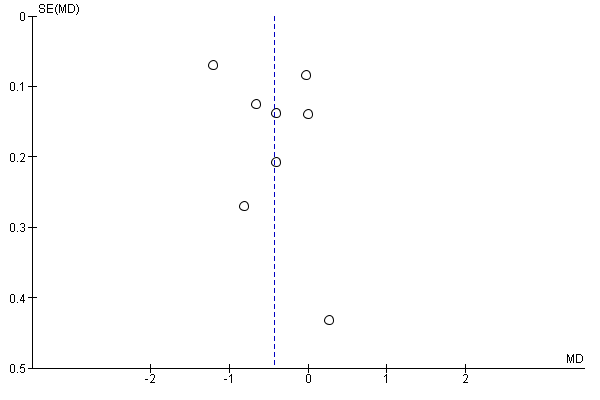


Figure S12. Funnel plot showing study precision against the MD effect estimate with 95% CIs for urinary pH. SE = Standard error.


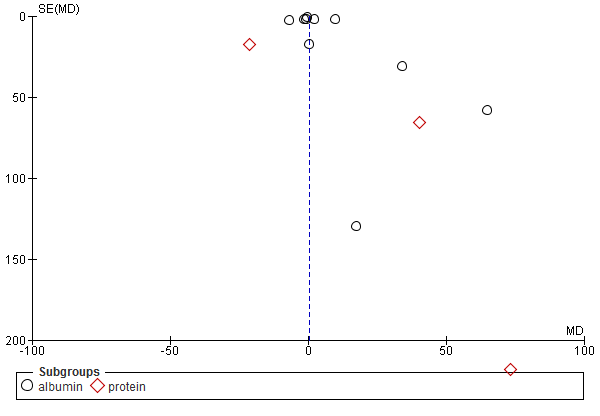


Figure S13. Funnel plot showing study precision against the WMD effect estimate with 95% CIs for urinary albumin. SE = Standard error Figure S18.


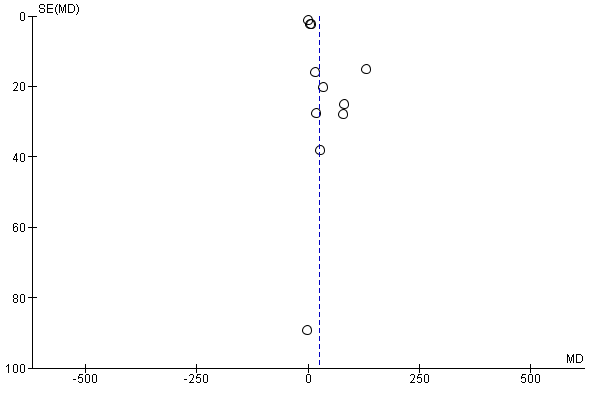
Figure S14. Funnel plot showing study precision against the MD effect estimate with 95% CIs for urinary calcium excretion. SE = Standard error.

| **Outcomes** | **No. of**  **Studies** | **Sample size** | **MD** | **95% CI** | **p-values** | **Inconsistency I^2^** |
| --- | --- | --- | --- | --- | --- | --- |
| GFR (ml/min/1.73m^2^) | 14 | 1324 | 4.96 | [3.38, 6.54] | <0.001 | 0% |
| Creatinine (µmol/l) | 16 | 1561 | -1.35 | [-4.22, 1.51] | 0.35 | 69% |
| Urea (mmol/l) | 9 | 762 | 1.06 | [0.76, 1.36] | <0.001 | 27% |
| Uric acid (µmol/l) | 3 | 152 | 0.19 | [-0.20, 0.57] | 0.35 | 0% |
| Urinary Albumin/protein (mg/24h) | 8 | 674 | -0.22 | [-1.73, 1.29] | 0.78 | 0% |
| Urinary calcium excretion (mg/24h) | 5 | 562 | 46.59 | [-11.26, 104.44] | 0.11 | 88% |

| **Outcomes** | **No. of**  **Studies** | **Sample size** | **MD** | **95% CI** | **p-values** | **Inconsistency I^2^** |
| --- | --- | --- | --- | --- | --- | --- |
| GFR (ml/min/1.73m^2^) | 15 | 1296 | 7.73 | [4.43, 11.04] | <0.001 | 63% |
| Creatinine (µmol/l) | 14 | 1205 | -2.64 | [-4.92, -0.35] | 0.02 | 61% |
| Urea (mmol/l) | 9 | 752 | 1.87 | [1.13, 2.61] | <0.001 | 92% |
| Uric acid (µmol/l) | 5 | 168 | 0.14 | [-0.26, 0.55] | 0.49 | 43% |
| Urinary pH | 5 | 170 | -0.46 | [-0.99, 0.08] | 0.09 | 96% |
| Urinary Albumin/protein (mg/24h) | 4 | 501 | 2.92 | [-3.23, 9.08] | 0.35 | 85% |
| Urinary calcium excretion (mg/24h) | 6 | 529 | 27.34 | [14.30, 40.38] | <0.001 | 94% |

Table S1: Sensitivity analysis for subjects without T2D: Pooled estimates of effect size (95% confidence intervals) expressed as weighted mean difference for the effects of HP vs. NP/LP diets on outcomes of renal function.

Table S2: Sensitivity analysis for obese subjects: Pooled estimates of effect size (95% confidence intervals) expressed as weighted mean difference for the effects of HP vs. NP/LP diets on outcomes of renal function.

| **Outcomes** | **No. of**  **Studies** | **Sample size** | **MD** | **95% CI** | **p-values** | **Inconsistency I^2^** |
| --- | --- | --- | --- | --- | --- | --- |
| GFR (ml/min/1.73m^2^) | 12 | 992 | 6.23 | [3.52, 8.93] | <0.001 | 0% |
| Creatinine (µmol/l) | 14 | 1236 | -1.68 | [-5.14, 1.77] | 0.34 | 67% |
| Urea (mmol/l) | 7 | 730 | 1.03 | [0.75, 1.30] | <0.00001 | 20% |
| Uric acid (µmol/l) | 3 | 151 | 0.18 | [-0.22, 0.58] | 0.37 | 0% |
| Urinary Albumin/protein (mg/24h) | 6 | 618 | -0.04 | [-1.68, 1.60] | 0.96 | 0% |
| Urinary calcium excretion (mg/24h) | 4 | 546 | 50.69 | [-10.49, 111.88] | 0.10 | 91% |

Table S3: Sensitivity analysis for long-term studies (≥12 weeks): Pooled estimates of effect size (95% confidence intervals) expressed as weighted mean difference for the effects of HP vs. NP/LP diets on outcomes of renal function.

| **Outcomes** | **No. of**  **Studies** | **Sample size** | **MD** | **95% CI** | **p-values** | **Inconsistency I^2^** |
| --- | --- | --- | --- | --- | --- | --- |
| GFR (ml/min/1.73m^2^) | 6 | 303 | 4.55 | [0.11, 8.99] | 0.04 | 0% |
| Creatinine (µmol/l) | 8 | 784 | 0.59 | [-2.31, 3.48] | 0.69 | 22% |
| Urea (mmol/l) | 3 | 71 | 1.47 | [0.07, 2.88] | 0.04 | 32% |
| Uric acid (µmol/l) | 2 | 40 | 0.22 | [-0.52, 0.96] | 0.56 | 0% |
| Urinary Albumin/protein (mg/24h) | 7 | 282 | -2.09 | [-4.51, 0.33] | 0.09 | 0% |
| Urinary calcium excretion (mg/24h) | 4 | 179 | 17.04 | [-8.33, 42.40] | 0.19 | 0% |

Table S4: Sensitivity analysis for T2D subjects: Pooled estimates of effect size (95% confidence intervals) expressed as weighted mean difference for the effects of HP vs. NP/LP diets on outcomes of renal function.

**References S1-32**

[[1-32](#_ENREF_1)]

1. Skov AR, Toubro S, Bulow J, Krabbe K, Parving HH, et al. (1999) Changes in renal function during weight loss induced by high vs low-protein low-fat diets in overweight subjects. Int J Obes Relat Metab Disord 23: 1170-1177.

2. Jenkins DJ, Kendall CW, Vidgen E, Augustin LS, van Erk M, et al. (2001) High-protein diets in hyperlipidemia: effect of wheat gluten on serum lipids, uric acid, and renal function. Am J Clin Nutr 74: 57-63.

3. Brinkworth GD, Buckley JD, Noakes M, Clifton PM (2010) Renal function following long-term weight loss in individuals with abdominal obesity on a very-low-carbohydrate diet vs high-carbohydrate diet. J Am Diet Assoc 110: 633-638.

4. Ferrara LA, Innelli P, Palmieri V, Limauro S, De Luca G, et al. (2006) Effects of different dietary protein intakes on body composition and vascular reactivity. Eur J Clin Nutr 60: 643-649.

5. Wagner EA, Falciglia GA, Amlal H, Levin L, Soleimani M (2007) Short-term exposure to a high-protein diet differentially affects glomerular filtration rate but not Acid-base balance in older compared to younger adults. J Am Diet Assoc 107: 1404-1408.

6. Leidy HJ, Carnell NS, Mattes RD, Campbell WW (2007) Higher protein intake preserves lean mass and satiety with weight loss in pre-obese and obese women. Obesity (Silver Spring) 15: 421-429.

7. Stern L, Iqbal N, Seshadri P, Chicano KL, Daily DA, et al. (2004) The effects of low-carbohydrate versus conventional weight loss diets in severely obese adults: one-year follow-up of a randomized trial. Ann Intern Med 140: 778-785.

8. Brinkworth GD, Noakes M, Keogh JB, Luscombe ND, Wittert GA, et al. (2004) Long-term effects of a high-protein, low-carbohydrate diet on weight control and cardiovascular risk markers in obese hyperinsulinemic subjects. Int J Obes Relat Metab Disord 28: 661-670.

9. Friedman AN, Ogden LG, Foster GD, Klein S, Stein R, et al. (2012) Comparative effects of low-carbohydrate high-protein versus low-fat diets on the kidney. Clin J Am Soc Nephrol 7: 1103-1111.

10. Gross JL, Zelmanovitz T, Moulin CC, De Mello V, Perassolo M, et al. (2002) Effect of a chicken-based diet on renal function and lipid profile in patients with type 2 diabetes: a randomized crossover trial. Diabetes Care 25: 645-651.

11. Krebs JD, Elley CR, Parry-Strong A, Lunt H, Drury PL, et al. (2012) The Diabetes Excess Weight Loss (DEWL) Trial: a randomised controlled trial of high-protein versus high-carbohydrate diets over 2 years in type 2 diabetes. Diabetologia 55: 905-914.

12. Larsen RN, Mann NJ, Maclean E, Shaw JE (2011) The effect of high-protein, low-carbohydrate diets in the treatment of type 2 diabetes: a 12 month randomised controlled trial. Diabetologia 54: 731-740.

13. Cao JJ, Johnson LK, Hunt JR (2011) A diet high in meat protein and potential renal acid load increases fractional calcium absorption and urinary calcium excretion without affecting markers of bone resorption or formation in postmenopausal women. J Nutr 141: 391-397.

14. Luscombe-Marsh ND, Noakes M, Wittert GA, Keogh JB, Foster P, et al. (2005) Carbohydrate-restricted diets high in either monounsaturated fat or protein are equally effective at promoting fat loss and improving blood lipids. Am J Clin Nutr 81: 762-772.

15. Noakes M, Keogh JB, Foster PR, Clifton PM (2005) Effect of an energy-restricted, high-protein, low-fat diet relative to a conventional high-carbohydrate, low-fat diet on weight loss, body composition, nutritional status, and markers of cardiovascular health in obese women. Am J Clin Nutr 81: 1298-1306.

16. Li Z, Treyzon L, Chen S, Yan E, Thames G, et al. (2010) Protein-enriched meal replacements do not adversely affect liver, kidney or bone density: an outpatient randomized controlled trial. Nutr J 9: 72.

17. Velazquez Lopez L, Sil Acosta MJ, Goycochea Robles MV, Torres Tamayo M, Castaneda Limones R (2008) Effect of protein restriction diet on renal function and metabolic control in patients with type 2 diabetes: a randomized clinical trial. Nutr Hosp 23: 141-147.

18. Westman EC, Yancy WS, Jr., Mavropoulos JC, Marquart M, McDuffie JR (2008) The effect of a low-carbohydrate, ketogenic diet versus a low-glycemic index diet on glycemic control in type 2 diabetes mellitus. Nutr Metab (Lond) 5: 36.

19. Sargrad KR, Homko C, Mozzoli M, Boden G (2005) Effect of high protein vs high carbohydrate intake on insulin sensitivity, body weight, hemoglobin A1c, and blood pressure in patients with type 2 diabetes mellitus. J Am Diet Assoc 105: 573-580.

20. Liu X, Zhang G, Ye X, Li H, Chen X, et al. (2013) Effects of a low-carbohydrate diet on weight loss and cardiometabolic profile in Chinese women: a randomised controlled feeding trial. Br J Nutr: 1-10.

21. Nuttall FQ, Gannon MC, Saeed A, Jordan K, Hoover H (2003) The metabolic response of subjects with type 2 diabetes to a high-protein, weight-maintenance diet. J Clin Endocrinol Metab 88: 3577-3583.

22. Roughead ZK, Johnson LK, Lykken GI, Hunt JR (2003) Controlled high meat diets do not affect calcium retention or indices of bone status in healthy postmenopausal women. J Nutr 133: 1020-1026.

23. Johnston CS, Tjonn SL, Swan PD (2004) High-protein, low-fat diets are effective for weight loss and favorably alter biomarkers in healthy adults. J Nutr 134: 586-591.

24. Pomerleau J, Verdy M, Garrel DR, Nadeau MH (1993) Effect of protein intake on glycaemic control and renal function in type 2 (non-insulin-dependent) diabetes mellitus. Diabetologia 36: 829-834.

25. Frank H, Graf J, Amann-Gassner U, Bratke R, Daniel H, et al. (2009) Effect of short-term high-protein compared with normal-protein diets on renal hemodynamics and associated variables in healthy young men. Am J Clin Nutr 90: 1509-1516.

26. Wycherley TP BG, Clifton PM, Noakes M (2012) Comparison of the effects of 52 weeks weight loss with either a high-protein or high-carbohydrate diet on body composition and cardiometabolic risk factors in overweight and obese males. Nutrition and Diabetes 2.

27. Nuttall FQ, Gannon MC (2006) The metabolic response to a high-protein, low-carbohydrate diet in men with type 2 diabetes mellitus. Metabolism 55: 243-251.

28. Gannon MC, Nuttall FQ, Saeed A, Jordan K, Hoover H (2003) An increase in dietary protein improves the blood glucose response in persons with type 2 diabetes. Am J Clin Nutr 78: 734-741.

29. Yancy WS, Jr., Olsen MK, Dudley T, Westman EC (2007) Acid-base analysis of individuals following two weight loss diets. Eur J Clin Nutr 61: 1416-1422.

30. Luger M, Holstein B, Schindler K, Kruschitz R, Ludvik B (2013) Feasibility and efficacy of an isocaloric high-protein vs. standard diet on insulin requirement, body weight and metabolic parameters in patients with type 2 diabetes on insulin therapy. Exp Clin Endocrinol Diabetes 121: 286-294.

31. Jesudason DR, Pedersen E, Clifton PM (2013) Weight-loss diets in people with type 2 diabetes and renal disease: a randomized controlled trial of the effect of different dietary protein amounts. Am J Clin Nutr 98: 494-501.

32. Juraschek SP, Appel LJ, Anderson CA, Miller ER, 3rd (2013) Effect of a high-protein diet on kidney function in healthy adults: results from the OmniHeart trial. Am J Kidney Dis 61: 547-554.
