# Supplementary material for: Comparison of High vs. Normal/Low Protein Diets on Renal Function in Subjects without Chronic Kidney Disease: A Systematic Review and Meta-Analysis
Source: PLoS One. 2014 May 22;9(5):e97656. doi: 10.1371/journal.pone.0097656 (PMC4031217; doi:10.1371/journal.pone.0097656)
Supplement: References S1 — (DOCX) [file pone.0097656.s020.docx]

**References S1-32**

[[1-32](#_ENREF_1)]

1. Skov AR, Toubro S, Bulow J, Krabbe K, Parving HH, et al. (1999) Changes in renal function during weight loss induced by high vs low-protein low-fat diets in overweight subjects. Int J Obes Relat Metab Disord 23: 1170-1177.

2. Jenkins DJ, Kendall CW, Vidgen E, Augustin LS, van Erk M, et al. (2001) High-protein diets in hyperlipidemia: effect of wheat gluten on serum lipids, uric acid, and renal function. Am J Clin Nutr 74: 57-63.

3. Brinkworth GD, Buckley JD, Noakes M, Clifton PM (2010) Renal function following long-term weight loss in individuals with abdominal obesity on a very-low-carbohydrate diet vs high-carbohydrate diet. J Am Diet Assoc 110: 633-638.

4. Ferrara LA, Innelli P, Palmieri V, Limauro S, De Luca G, et al. (2006) Effects of different dietary protein intakes on body composition and vascular reactivity. Eur J Clin Nutr 60: 643-649.

5. Wagner EA, Falciglia GA, Amlal H, Levin L, Soleimani M (2007) Short-term exposure to a high-protein diet differentially affects glomerular filtration rate but not Acid-base balance in older compared to younger adults. J Am Diet Assoc 107: 1404-1408.

6. Leidy HJ, Carnell NS, Mattes RD, Campbell WW (2007) Higher protein intake preserves lean mass and satiety with weight loss in pre-obese and obese women. Obesity (Silver Spring) 15: 421-429.

7. Stern L, Iqbal N, Seshadri P, Chicano KL, Daily DA, et al. (2004) The effects of low-carbohydrate versus conventional weight loss diets in severely obese adults: one-year follow-up of a randomized trial. Ann Intern Med 140: 778-785.

8. Brinkworth GD, Noakes M, Keogh JB, Luscombe ND, Wittert GA, et al. (2004) Long-term effects of a high-protein, low-carbohydrate diet on weight control and cardiovascular risk markers in obese hyperinsulinemic subjects. Int J Obes Relat Metab Disord 28: 661-670.

9. Friedman AN, Ogden LG, Foster GD, Klein S, Stein R, et al. (2012) Comparative effects of low-carbohydrate high-protein versus low-fat diets on the kidney. Clin J Am Soc Nephrol 7: 1103-1111.

10. Gross JL, Zelmanovitz T, Moulin CC, De Mello V, Perassolo M, et al. (2002) Effect of a chicken-based diet on renal function and lipid profile in patients with type 2 diabetes: a randomized crossover trial. Diabetes Care 25: 645-651.

11. Krebs JD, Elley CR, Parry-Strong A, Lunt H, Drury PL, et al. (2012) The Diabetes Excess Weight Loss (DEWL) Trial: a randomised controlled trial of high-protein versus high-carbohydrate diets over 2 years in type 2 diabetes. Diabetologia 55: 905-914.

12. Larsen RN, Mann NJ, Maclean E, Shaw JE (2011) The effect of high-protein, low-carbohydrate diets in the treatment of type 2 diabetes: a 12 month randomised controlled trial. Diabetologia 54: 731-740.

13. Cao JJ, Johnson LK, Hunt JR (2011) A diet high in meat protein and potential renal acid load increases fractional calcium absorption and urinary calcium excretion without affecting markers of bone resorption or formation in postmenopausal women. J Nutr 141: 391-397.

14. Luscombe-Marsh ND, Noakes M, Wittert GA, Keogh JB, Foster P, et al. (2005) Carbohydrate-restricted diets high in either monounsaturated fat or protein are equally effective at promoting fat loss and improving blood lipids. Am J Clin Nutr 81: 762-772.

15. Noakes M, Keogh JB, Foster PR, Clifton PM (2005) Effect of an energy-restricted, high-protein, low-fat diet relative to a conventional high-carbohydrate, low-fat diet on weight loss, body composition, nutritional status, and markers of cardiovascular health in obese women. Am J Clin Nutr 81: 1298-1306.

16. Li Z, Treyzon L, Chen S, Yan E, Thames G, et al. (2010) Protein-enriched meal replacements do not adversely affect liver, kidney or bone density: an outpatient randomized controlled trial. Nutr J 9: 72.

17. Velazquez Lopez L, Sil Acosta MJ, Goycochea Robles MV, Torres Tamayo M, Castaneda Limones R (2008) Effect of protein restriction diet on renal function and metabolic control in patients with type 2 diabetes: a randomized clinical trial. Nutr Hosp 23: 141-147.

18. Westman EC, Yancy WS, Jr., Mavropoulos JC, Marquart M, McDuffie JR (2008) The effect of a low-carbohydrate, ketogenic diet versus a low-glycemic index diet on glycemic control in type 2 diabetes mellitus. Nutr Metab (Lond) 5: 36.

19. Sargrad KR, Homko C, Mozzoli M, Boden G (2005) Effect of high protein vs high carbohydrate intake on insulin sensitivity, body weight, hemoglobin A1c, and blood pressure in patients with type 2 diabetes mellitus. J Am Diet Assoc 105: 573-580.

20. Liu X, Zhang G, Ye X, Li H, Chen X, et al. (2013) Effects of a low-carbohydrate diet on weight loss and cardiometabolic profile in Chinese women: a randomised controlled feeding trial. Br J Nutr: 1-10.

21. Nuttall FQ, Gannon MC, Saeed A, Jordan K, Hoover H (2003) The metabolic response of subjects with type 2 diabetes to a high-protein, weight-maintenance diet. J Clin Endocrinol Metab 88: 3577-3583.

22. Roughead ZK, Johnson LK, Lykken GI, Hunt JR (2003) Controlled high meat diets do not affect calcium retention or indices of bone status in healthy postmenopausal women. J Nutr 133: 1020-1026.

23. Johnston CS, Tjonn SL, Swan PD (2004) High-protein, low-fat diets are effective for weight loss and favorably alter biomarkers in healthy adults. J Nutr 134: 586-591.

24. Pomerleau J, Verdy M, Garrel DR, Nadeau MH (1993) Effect of protein intake on glycaemic control and renal function in type 2 (non-insulin-dependent) diabetes mellitus. Diabetologia 36: 829-834.

25. Frank H, Graf J, Amann-Gassner U, Bratke R, Daniel H, et al. (2009) Effect of short-term high-protein compared with normal-protein diets on renal hemodynamics and associated variables in healthy young men. Am J Clin Nutr 90: 1509-1516.

26. Wycherley TP BG, Clifton PM, Noakes M (2012) Comparison of the effects of 52 weeks weight loss with either a high-protein or high-carbohydrate diet on body composition and cardiometabolic risk factors in overweight and obese males. Nutrition and Diabetes 2.

27. Nuttall FQ, Gannon MC (2006) The metabolic response to a high-protein, low-carbohydrate diet in men with type 2 diabetes mellitus. Metabolism 55: 243-251.

28. Gannon MC, Nuttall FQ, Saeed A, Jordan K, Hoover H (2003) An increase in dietary protein improves the blood glucose response in persons with type 2 diabetes. Am J Clin Nutr 78: 734-741.

29. Yancy WS, Jr., Olsen MK, Dudley T, Westman EC (2007) Acid-base analysis of individuals following two weight loss diets. Eur J Clin Nutr 61: 1416-1422.

30. Luger M, Holstein B, Schindler K, Kruschitz R, Ludvik B (2013) Feasibility and efficacy of an isocaloric high-protein vs. standard diet on insulin requirement, body weight and metabolic parameters in patients with type 2 diabetes on insulin therapy. Exp Clin Endocrinol Diabetes 121: 286-294.

31. Jesudason DR, Pedersen E, Clifton PM (2013) Weight-loss diets in people with type 2 diabetes and renal disease: a randomized controlled trial of the effect of different dietary protein amounts. Am J Clin Nutr 98: 494-501.

32. Juraschek SP, Appel LJ, Anderson CA, Miller ER, 3rd (2013) Effect of a high-protein diet on kidney function in healthy adults: results from the OmniHeart trial. Am J Kidney Dis 61: 547-554.
